# Traffic condition prediction for highway within work zones under dynamic traffic organization changes

**DOI:** 10.1371/journal.pone.0351729

**Published:** 2026-06-18

**Authors:** Feiping Xu, Bohan Liu, Hang Liu, Yonghao Wang, Jundong Wang, Chengcheng Wang

**Affiliations:** 1 Shandong Hi-Speed Company Limited, Jinan, China; 2 Beijing Key Laboratory of Traffic Engineering, College of Metropolitan Transportation, Beijing University of Technology, Beijing, China; 3 Shandong Provincial Communications Planning and Design Institute Group Co., Ltd, Jinan, China; 4 Shandong Hi-speed Group Innovation Research Institute, Jinan, China; 5 State Key Lab of Intelligent Transportation System, Jinan, China; 6 Central South University School of Traffic & Transportation Engineering, Changsha, China; National University of Defense Technology, CHINA

## Abstract

In the context of sustainable transportation development, reducing carbon emissions, energy waste, and noise pollution caused by traffic congestion has become an urgent task for achieving environmental and social sustainability. The key to this goal lies in mitigating and preventing traffic congestion, for which high accuracy traffic condition prediction models serve as essential tools. During the reconstruction and expansion of highways and urban arterial roads, frequent adjustments to traffic organization and changes in geometric alignment introduce dynamic and uncertain characteristics into the traffic system. Existing methods struggle to accurately predict traffic conditions in the modified sections. To address this challenge, this study proposes a Dynamic Bayesian Graph Convolutional Neural Network (DBGCN). The model incorporates road geometric parameters and dynamic traffic organization changes as key inputs. It employs a Dynamic Bayesian Network (DBN) to model multi-source dynamic information and infer a dynamic adjacency matrix that reflects latent spatiotemporal dependencies between nodes. This dynamic adjacency matrix is then input into a Graph Convolutional Network (GCN), which fuses spatiotemporal features with traffic flow data to achieve accurate traffic conditions prediction for upgraded sections. Validation on the Wuxuan highway demonstrates that the proposed method outperforms benchmark models in traffic conditions prediction accuracy and produces traffic conditions propagation diagrams with high interpretability.

## 1 Introduction

With the acceleration of urbanization, traffic congestion has emerged as a global challenge that restricts sustainable urban development and impedes improvements in residents’ quality of life. To alleviate the growing pressure on transportation networks, the expansion and renovation of existing highways and major urban arterial roads are widely adopted engineering strategies. However, although these projects mitigate long term congestion, the complex traffic management measures implemented during construction—such as lane closures, temporary diversions, and speed restrictions—together with phased modifications in road geometry (e.g., new ramps, widened sections, optimized alignments)—place the transportation system in a highly dynamic and uncertain state, wherein accurately detecting and tracking evolving signal structures is a fundamental challenge [1]. This dual dynamism often induces traffic flow disruptions, leading to frequent and pronounced fluctuations in traffic conditions levels. Consequently, the upgraded sections frequently emerge as hotspots of inefficient traffic operations. Therefore, accurate and dynamic prediction of real time traffic conditions in these areas is of critical importance for optimizing traffic management during construction, improving network efficiency, and ensuring road safety.

In recent years, traffic state prediction technology has made significant progress. Early research was predominantly model driven, typically requiring simulations to study the propagation and evolution of traffic congestion. From the perspective of specific simulation methods, these can be categorized into macro models and micro models. Microscopic models focus on individual vehicle behavior, transforming traffic congestion into a queueing theory problem. For instance, Morales et al. [2] estimated queue lengths caused by traffic events using arrival and departure rates; Newell et al. [3] proposed a dynamic queueing model based on traveling wave theory, validating its applicability to real world traffic conditions; Helbing et al. [4] studied traffic flow at intersections, proposing a flow analysis method based on dynamic queuing; additionally, Zheng et al. [5] estimated the Hurst parameter for peak hour traffic using a queuing theory model, indicating strong self-similarity in the queuing process at road nodes. However, the simplification of road networks in micro-level models makes them difficult to apply in large scale traffic networks.

Macroscopic models, such as the LWR model and CTM, describe the spatiotemporal evolution of traffic congestion through the analysis of aggregate parameters such as flow, density, and speed. Lighthill and Whitham [6], along with Richards [7], first proposed the continuous LWR traffic flow model. Building on this foundation, Daganzo [8] introduced the Cell Transmission Model (CTM), which partitions roads into multiple cells and characterizes the propagation of traffic states by solving variables such as flow and density. Consequently, it has been widely applied to studies of both periodic and non-periodic congestion. Furthermore, to address uncertainty in congestion propagation, Wang et al. [9] introduced the Susceptible–Infected–Susceptible (SIS) model. By integrating it with CTM, they developed the SIS–CTM model, which probabilistically characterizes the transition and diffusion of road traffic conditions, enabling more accurate simulation of congestion dynamics. Yang et al. [10] integrated intersection traffic flow models with road network topology models to achieve real time prediction of macroscopic propagation characteristics in network traffic flows. However, although traditional theoretical models effectively capture congestion propagation mechanisms, they are fundamentally grounded in traffic flow theory. Their application to large scale road networks necessitates extensive data collection and parameter calibration, which poses significant challenges for practical implementation.

With the rapid advancement of artificial intelligence, data driven models have increasingly emerged as a research focus for predicting traffic congestion propagation. Traditional time series models [11], machine learning methods [12] and deep learning architectures such as Convolutional Neural Networks [13] and Deep Neural Networks [14] primarily rely on historical data, which limits their ability to effectively capture complex spatiotemporal dependencies. Graph neural networks (GNNs), particularly graph convolutional networks (GCNs), show substantial potential for traffic state prediction owing to their strong spatial feature extraction capabilities. Yu et al. [15] proposed spatiotemporal graph convolutional networks (STGCN) to capture variations in traffic flows, whereas Defferrard et al. [16] incorporated graph convolutional layers and convolutional sequence learning layers into convolutional modules to improve the ability of models to capture dynamic changes in traffic flows. However, a critical limitation of existing GNN-based methods is their reliance on predefined static adjacency matrices (e.g., fixed Euclidean distances or historical origin–destination matrices) to represent the spatial topology of road networks. This assumption breaks down in expansion and reconstruction scenarios. When road alignment and traffic organization change dynamically, the actual spatial dependencies among nodes also evolve. Static adjacency matrices cannot reflect this dynamic topology, thereby limiting model performance in predicting traffic states. Although some studies attempt to mitigate this issue by learning implicit dynamic weights, such methods often lack explicit modeling of the fundamental physical factors that drive topological changes—such as alignment modifications or traffic organization adjustments. As a result, the interpretability of the learned relationships is limited, which hinders the integration of prior engineering knowledge.

In parallel, recent studies have increasingly recognized the need for dynamic graph representations in traffic forecasting. For instance, Ning et al. [17] proposed a Spatial–Temporal Causal Fusion Graph Neural Network (STCFGNN) that leverages Granger causality to infer time-varying spatial dependencies under traffic disruptions; Fan et al. [18] introduced DynaKey-GNN, a dynamic key node multigraph framework that adapts graph structures in response to volatile traffic conditions; and Theodoropoulos et al. [19] developed WEST GCN-LSTM, which incorporates regional connectivity policies—such as shared borders and adjustable hops—into spatiotemporal graph construction. While these approaches advance beyond static topologies, they remain largely data-driven and do not explicitly integrate domain-specific physical factors—such as road alignment changes or traffic organization adjustments—that directly govern topological evolution in construction zones. This gap motivates our design of a physically informed dynamic graph learning framework.

To address these challenges, this study proposes an innovative traffic conditions prediction technique that integrates road alignment metrics with dynamic traffic organization information to adaptively learn and predict traffic state evolution in upgraded road sections. The core concept is that changes in road alignment and traffic organization fundamentally drive the dynamic evolution of spatial dependency structures within road networks. To this end, we introduce the Dynamic Bayesian Graph Convolutional Network (DBGCN). The model first employs a Dynamic Bayesian Network (DBN) to represent multi-source dynamic information—including real-time or phased geometric parameters and traffic organization data—thereby learning and inferring a dynamic adjacency matrix that captures latent spatial dependencies among nodes [20]. The strength of the DBN lies in its ability to capture conditional probability dependencies and latent causal relationships among variables, ensuring that the generated adjacency matrix is not only dynamically adaptive but also retains clear physical meaning and interpretability. Subsequently, the adjacency matrix inferred by the DBN is used as input to a Graph Convolutional Network (GCN), replacing the traditional static adjacency matrix. Guided by this dynamic topology, the GCN effectively fuses spatiotemporal features with real-time traffic flow data, thereby enabling accurate traffic conditions prediction.

The main contributions of this study can be summarized as follows: (1) For the first time, road alignment metrics and dynamic traffic organization changes are systematically incorporated as key inputs for constructing a traffic conditions prediction model for road sections under expansion or reconstruction; (2) A DBN–GCN fusion architecture is proposed, which leverages a DBN to explicitly learn dynamic adjacency matrices from physical factors, thereby addressing the challenge of defining topology in dynamic scenarios for GCNs; (3) The learned adjacency matrix demonstrates strong physical interpretability, facilitating understanding of how engineering modifications affect spatial correlations in traffic flow; and (4) Validation on real-world cases shows that the proposed method outperforms existing benchmark models in traffic conditions prediction accuracy. The remainder of this paper is organized as follows: Section 2 presents the DBGCN model framework; Section 3 describes the data sources; Section 4 discusses baseline models, parameter selection, and results; and Section 5 concludes the paper and outlines directions for future research.

## 2 Methods

**Fig 1:**
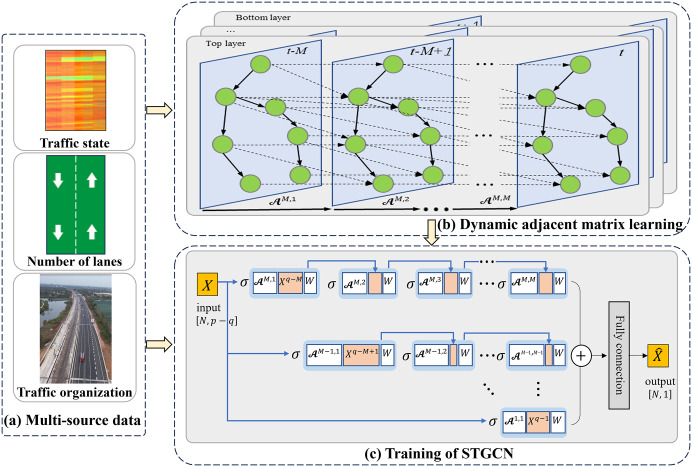
DBGCN model framework.

To address the challenges of predicting traffic conditions under dynamic changes in road alignment parameters and traffic organization during construction on expanded or reconstructed road sections, this study proposes a Dynamic Bayesian Graph Convolutional Network (DBGCN) to learn traffic state networks (Fig 1). The core approach explicitly incorporates dynamic adjustments to traffic organization (e.g., lane closures, diversion plan changes) into the graph construction process to more accurately capture spatial dependencies among road network nodes under expansion or reconstruction conditions. Specifically, this study adopts a two-stage modeling paradigm: first, network structure learning is conducted. A Dynamic Bayesian Network (DBN) is employed to analyze statistical dependencies between road geometric parameters and traffic flow data. Through a whitelist/blacklist mechanism, critical traffic organization changes are incorporated as prior constraints, guiding the DBN to learn an adjacency matrix that reflects the current traffic management state. Subsequently, net-work parameter learning is conducted. The dynamic adjacency matrix generated by the DBN, which integrates traffic organization information, is then used as the graph structure input. Graph Convolutional Networks (GCNs) are employed for spatiotemporal feature extraction, ultimately enabling the prediction of traffic conditions in the modified and expanded road sections.

### 2.1 Data preparation

**Fig 2:**
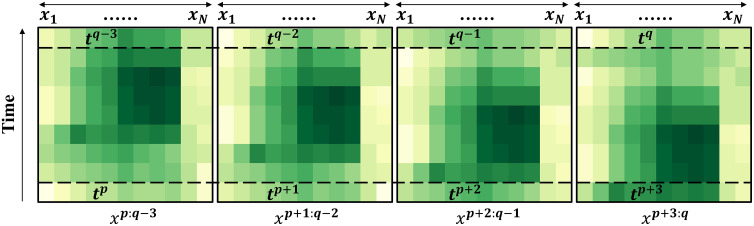
Example of the new dataset with a delay of 3 times (M = 3).

Based This study adopts travel speed as an indicator to characterize traffic conditions. To examine how earlier traffic states influence current conditions, a time-lag mechanism was introduced during dataset construction: when the time lag increases by one, the dataset’s time window shifts forward by one step. As illustrated in the dataset below, the samples span from time point 𝑝 to time point 𝑞 When the time lag is set to 𝑀, the dataset is divided into 𝑀 + 1 sliding subsets. For example, when 𝑀 = 3, the dataset structure transforms as shown in Fig 2. The dataset dimensions change from the original (q−p)×N to (q−p−3)×(4*N). In this new structure, each row reflects the evolution of a road segment’s state over four consecutive time points, thereby facilitating the second module’s learning of dynamic relationships among segments.

### 2.2 Network architecture learning

The adjacency matrix represents the transportation network and essentially functions as a Directed Acyclic Graph (DAG). However, changes in road alignment and traffic organization during construction can alter the correlation characteristics of traffic conditions among road segments. Consequently, the adjacency matrix cannot be treated as a fixed network topology. To address this issue, and guided by a data-driven approach, this study employs a Dynamic Bayesian Network (DBN) to infer adjacency relationships among road segments influenced by changes in road alignment and traffic organization, as illustrated in Fig 1(b). The following section details the congestion propagation structure learning process, illustrated through a two-time-slice DAG example.

Step 1: Constrain directed causal relationships between nodes by integrating empirical knowledge with the impacts of road construction. During congestion propagation, two natural constraints apply: (1) Due to road geometry or changes in traffic organization, once congestion occurs, it propagates only from the source node to its upstream neighboring nodes; (2) The current state of a node is influenced by its previous state, implying that nodes are self-connected.

Moreover, considering that expansion or reconstruction work may lead to road closures, the affected road segments completely lose their traffic functionality during closure, thereby nullifying the traffic influence relationship between these closed segments and their upstream segments.

Therefore, in any DAG spanning two time slices, the adjacency matrix is predefined as follows:


$$\begin{array}{c} \begin{array}{c} \left\{ \begin{array}{cc} & A_{i,i}=\text{1},\text{ if node }i\text{ has temporal self}-\text{dependence }(\text{self}-\text{connection})\\ & A_{i,j}=\text{0},\text{ if node }j\text{ is a downstream neighbor of node }i\\ & A_{i,k}\equiv \text{0},\text{if node }k\text{ is an upstream neighbor of node }i\text{ and is under construction} \end{array}\right.\; \end{array} \end{array}$$ (1)

Step 2: Construct a scoring function to determine the optimal structure. The optimal structure is essentially defined as a union of multiple parent–child node pairs ⟨j,i⟩, where j≠k*.* For each node pair ⟨j,i⟩*,* given the potential non-uniqueness of parent node j , the stochastic process by which$\;x_{j}$ influences $x_{i}$ is modeled using a multivariate Gaussian distribution. Therefore, given the observed data $\overset{m}{\underset{i,j}{D}}$, the conditional probability distribution for node pair ⟨j,i⟩, can be expressed as:


$$\begin{array}{c} \begin{array}{c} \text{P}\left(x_{i},x_{j}|\overset{m}{\underset{i,j}{D}}\right)=(\text{2}\pi {)}^{-\frac{N}{\text{2}}}|C{|}^{-\frac{\text{1}}{\text{2}}}\text{exp}\left(\frac{\overset{m}{\underset{i}{D}}C^{-\text{1}}{\left(\overset{m}{\underset{i}{D}}\right)}^{T}}{-\text{2}}\right) \end{array} \end{array}$$ (2)

Here, $C=Cov\left(\overset{M,m}{\underset{i,j}{D}}\right)$ denotes the covariance of the road segment data, and  N is given by q−M−p+1.

Given the observed dataset $\;D^{m}$, the scoring function corresponding to a network structure$\;G^{m}$ is defined as the joint probability of all node pairs, which can be expressed as:


$$\begin{array}{c} \begin{array}{c} Score\left(G^{m}|D^{m}\right)=\frac{\text{P}\left(G^{m}\right)\text{P}\left(D^{m}|G^{m}\right)}{\text{P}\left(D^{m}\right)}=\frac{\text{P}\left(G^{m}\right)}{\text{P}\left(D^{m}\right)}\prod_{i}P\left(x_{i},x_{j}|\overset{m}{\underset{i,j}{D}}\right) \end{array} \end{array}$$ (3)

Since each structure $\;G^{m}$ is assumed to be equally likely, the term $\frac{\text{P}\left(G^{m}\right)}{\text{P}\left(D^{M,m}\right)}$ becomes a constant. Therefore, Equation (3) can be equivalently written as:


$$\begin{array}{c} \begin{array}{c} Score\left(G^{m}|D^{m}\right)=\prod_{i}P\left(x^{i},x^{j}|\overset{m}{\underset{i,j}{D}}\right) \end{array} \end{array}$$ (4)

Step 3: Once the scoring function is determined, network structure learning becomes a search problem. Subject to the constraints defined in Step 1, the max–min hill-climbing heuristic algorithm is employed to traverse all possible network structures. The structure with the highest score is identified as the optimal structure, denoted by $\;\overset{m}{\underset{o}{G}}$, which is then transformed into the adjacency matrix required for the GCN, as follows:


$$\begin{array}{c} \begin{array}{c} {\text{A}}^{m}=\overset{m}{\underset{o}{G}}=\underset{G^{m}}{argmax}\text{Score}\left(G^{m}|D^{m}\right) \end{array} \end{array}$$ (5)

It is worth noting that although the proposed framework supports dynamic inference of the adjacency matrix at each time step, traffic organization changes in highway reconstruction and expansion projects typically occur as planned, stage-wise events (e.g., lane closures or speed limit adjustments taking effect at predefined times). In practical deployment, the DBN-based structure learning can therefore be triggered only when a traffic management plan is updated—such as at the transition between construction phases—rather than executed at every time interval. This event-driven update strategy preserves the model’s ability to adapt to topological evolution while significantly reducing computational overhead, making it suitable for real-time applications.

### 2.3 Learning network parameters

This section leverages the learned graph structure by representing road segments with influence relationships as parent–child node pairs. A Spatiotemporal Graph Convolutional Network (STGCN) is introduced to quantify the degree of influence between parent–child nodes through a deep neural network framework.

To accommodate adjacency matrices at different levels, this study constructs a stacked architecture based on the STGCN, as illustrated in the red box of Fig 1(c). In this architecture, the number of layers in the STGCN structure corresponds to that of the stacked state propagation network. Within each layer, traffic states propagate downward through each STGCN module, facilitating information transmission. It is important to note that the time-delay parameter M is adjustable, allowing analysis of long-term effects on traffic states.

As an example at the top layer, when the time delay is set to M, the model selects data from the M preceding time steps before the current time step q as feature inputs. These features are then propagated downward through the STGCN at each time step, yielding the final feature output as:


$$\begin{array}{c} \begin{array}{c} \overset{t+\text{1}}{\underset{M}{𝐇}}=\prod_{m=\text{1}}^{M}\sigma A^{M,m}X^{t-M}W^{M,m} \end{array} \end{array}$$ (6)

$$\begin{array}{c} \begin{array}{c} {\hat{X}}^{t+\text{1}}=F\left(\omega ,\sum_{M}\overset{t+\text{1}}{\underset{M}{𝐇}}\right) \end{array} \end{array}$$ (7)

Finally, the feature outputs from all layers are summed to obtain $\;\sum_{M}\overset{t+\text{1}}{\underset{M}{𝐇}}$, and a fully connected layer is applied to map the result to the sample space, as follows:

Here, F(·) denotes the fully connected layer, which essentially performs a linear weighted summation, and  ω  represents the weights.

## 3. Data sources

The study area is located on the Wuhu–Xuancheng Expressway in China, specifically between the Wuxuan Interchange and the Wuhu Toll Station, and is divided into 164 road segments based on its topological structure. Before the expansion and renovation, the highway, consisted of four lanes in each direction, with a speed limit of 100 km/h. Construction was carried out in stages, extending from the Wuxuan Interchange to the Wuhu Toll Station. Within the construction zones, the number of lanes was reduced to two in each direction, and the speed limit was progressively lowered to 80 km/h, 60 km/h, and 40 km/h.

The data used in this study comprise traffic flow data and traffic organization schemes. Traffic flow data were obtained from AutoNavi Maps. Using API interfaces, data were collected for 164 topological road segments within the study area, covering 10-minute traffic flow intervals and per-minute average speeds from October 22 to November 10, 2024. Verification confirmed that the rate of missing data was below 1%. A small number of missing values were imputed using linear interpolation, thereby ensuring data integrity and quality. While it is acknowledged that linear interpolation may not fully capture abrupt dynamics during peak congestion periods, the extremely low missing rate—coupled with the fact that most gaps occurred during stable traffic conditions—suggests that the impact on overall traffic pattern representation and model performance is negligible.

**Fig 3:**
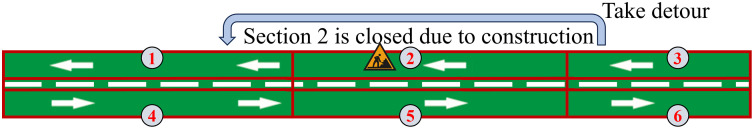
Example of Black and White List Settings.

The traffic organization plan—including road topology, lane counts, geometric parameters, lane closure arrangements, and speed limits—was derived from the official design and construction documents of the highway expansion project. These documents specify implementation details for each project phase. Based on the traffic organization design in the expansion plan, corresponding blacklists and whitelists were manually constructed for each construction phase to incorporate actual traffic arrangements into the model. The whitelist contains road segment connections permitted for direct passage, whereas the blacklist specifies connections prohibited due to closures or diversions. As shown in Fig 3, since the left side of the road is not under construction, normal upstream to downstream connections (e.g., Section 6 to Section 5, Section 5 to Section 4) are included in the whitelist, with no blacklist entries specified. However, because Section 2 on the right side is under construction, the connections from Section 1 to Section 2 and from Section 2 to Section 3 are designated as blacklist entries. This effectively incorporates the closure information for Section 2 into subsequent model iterations. The blacklists and whitelists are dynamically updated as construction progresses and are incorporated during network structure learning.

Ultimately, this study uses a strict temporal split: the first 24 consecutive days of data are used for training, and the last 6 days for testing. For each time step, the dataset includes actual speed values, lane counts, and speed limit values (Table 1). Among these, actual speed is a time-varying variable and serves as the model’s prediction target, whereas lane counts and speed limit values are adjusted according to construction progress but remain constant within the same construction phase.

**Table 1:** Dataset Format.

Field Name	Section 1	Section 2	Section 3	Section 4	Section 5	……
Actual Speed	84	76	48	74	89	……
Number of Lanes	2	1	1	1	2	……
Speed Limit	100	80	60	80	100	……

## 4. Results and case analysis

### 4.1. Baseline model

Since congestion indicators are derived from traffic speeds, congestion propagation under special circumstances (e.g., peak travel periods or traffic accidents) is inferred through traffic speed forecasting. Many existing studies focus on either congestion propagation or short-term traffic forecasting, employing diverse data structures and required data fields. Therefore, the DBGCN model proposed in this study cannot be directly compared with all previously developed models. The primary objective is to examine whether incorporating dynamically learned adjacency matrices that account for traffic organization changes can enhance the model’s inference capabilities. A secondary objective is to verify whether earlier traffic states influence current traffic conditions, which underpins the rationale for constructing a stacked model framework. Consequently, three representative models were selected as benchmarks:

(1) LSTM (Long Short-Term Memory Network): LSTM is a variant of recurrent neural networks (RNNs) specifically designed to address long-term dependency problems, making it well suited for processing time-series data. LSTMs retain historical information across multiple time steps, providing a distinct advantage in forecasting congestion indices over time. Because traffic flow and congestion evolution typically exhibit long-term dependencies, LSTMs effectively capture how past traffic conditions influence current and future congestion indices. Consequently, LSTMs are effective in modeling the spatiotemporal dynamics of traffic conditions when addressing sequential forecasting challenges in traffic congestion.
(2) Dynamic Bayesian Networks (DBNs): DBNs are directed probabilistic graphical models that apply probability theory to describe the propagation of traffic states between adjacent road segments. For continuous input features (e.g., speed), this propagation process is modeled using a Gaussian mixture model, in which child nodes infer their states by aggregating information from their parent nodes.
(3) STGCN (Spatio Temporal Graph Convolutional Neural Network): STGCN is a deep learning model that integrates graph convolutions with temporal convolutions, enabling the simultaneous modeling of spatial correlations and temporal evolution in traffic data. In this study, the STGCN leverages the topological structure of the road network. It extracts spatial dependencies among road segments through graph convolution while capturing temporal variations in traffic conditions via temporal convolution, thereby enabling accurate prediction of future traffic conditions. This model is particularly well suited for traffic scenarios with complex spatiotemporal dependencies and demonstrates strong expressive power and predictive performance.

### 4.2. Parameter settings

At the data input layer, as the time-lag value 𝑀 increases, traffic state information from earlier time slices is aggregated into the current traffic state. Given that the duration of traffic congestion is finite, the value of 𝑀 should not be set excessively high. In this study, 𝑀 is set to values of 1, 2, and 3. The MAE and RMSE metrics corresponding to each model under different values of M were calculated, as shown in Table 2. As the value of M increases, the errors of all four models show a gradual upward trend. This trend indicates that during congestion propagation, the traffic state of a given node is more strongly influenced by its first-order parent nodes in the temporal dimension, which is consistent with the first-order Markov property in Bayesian networks. In other words, the current traffic state of the studied object depends only on the most recent previous state. Therefore, all subsequent results are analyzed with M = 1.

**Table 2:** Comparison of Prediction Errors Across Models with Different Time-Lag Values.

Evaluation Metrics	time-lag value(M)	DBN	LSTM	STGCN	DBGCN
MAE	1	**2.4988**	**2.4334**	**2.4772**	**2.4112**
	2	2.5087	2.4820	2.4867	2.4594
	3	2.5137	2.5064	2.5015	2.4635
RMSE	1	**3.9255**	**3.8267**	**3.8819**	**3.8017**
	2	4.0047	3.9032	3.9595	3.8577
	3	4.0232	3.9215	3.9983	3.9057

During model training, this study employs the mean squared error (MSE) between actual and predicted values as the loss function. The Adam optimization algorithm is used to update the parameters of the graph convolutional network, which consists of 2 graph convolutional layers, with the learning rate set to10 − 3 to accelerate convergence. Early stopping is also applied to prevent overfitting. If the MSE does not improve by more than 10 − 6 over 10 consecutive iterations, the training process is terminated.

The model was trained on one month of historical data with a 10 minute temporal resolution. The input features included road topology, traffic conditions, and other relevant information, with an input stride of 6. For the output stride, we evaluated the performance of the model proposed in this paper using Mean Absolute Error (MAE), Root Mean Square Error (RMSE), and Mean Absolute Percentage Error (MAPE); the results are reported in Table 3. As the prediction horizon increases, prediction accuracy slightly decreases; however, all MAPE values remain below 0.05, indicating that the prediction accuracy consistently exceeds 95%. All subsequent analyses in the Results section are based on an output stride of 1, i.e., forecasting traffic conditions for the next 10 minutes using the preceding two hours of historical traffic data.

**Table 3:** Model Performance at Different Prediction Steps.

Prediction Step Size	1	2	3	4	5	6
MAE	2.411272	2.469494	2.50866	2.533829	2.572402	2.620706
RMSE	3.801724	3.892094	3.952044	4.000186	4.056624	4.120277
MAPE	0.04037	0.041734	0.042711	0.043359	0.044182	0.045116

### 4.3. Analysis of results

To validate the predictive performance of the proposed DBGCN model under dynamic variations in road alignment metrics and traffic organization, comparative experiments were conducted using the real highway dataset described above. Mean absolute error (MAE) and root mean square error (RMSE) were used to evaluate the performance of the congestion propagation inference model.

Table 4 presents the comparative results between the DBGCN model and several baseline models in terms of mean absolute error (MAE) and root mean square error (RMSE). As shown in Table 4, the proposed DBGCN model significantly outperforms all baseline models across both metrics, achieving the lowest MAE (2.4112) and RMSE (3.8017), thereby demonstrating a clear advantage in overall prediction accuracy. Notably, models such as DBN and STGCN, which incorporate spatial relationships, underperformed compared with the traditional time series forecasting model LSTM. This finding suggests that changes in traffic organization within the expansion area pose significant challenges for traditional models in learning spatial relationships. The proposed DBGCN model effectively addresses this limitation, thereby enhancing prediction accuracy.

**Table 4:** Performance Metrics of Different Models.

Model	Average MAE	Average RMSE
LSTM	2.4334	3.8267
DBN	2.4988	3.9255
STGCN	2.4772	3.8819
DBGCN	**2.4112**	**3.8017**

**Fig 4:**
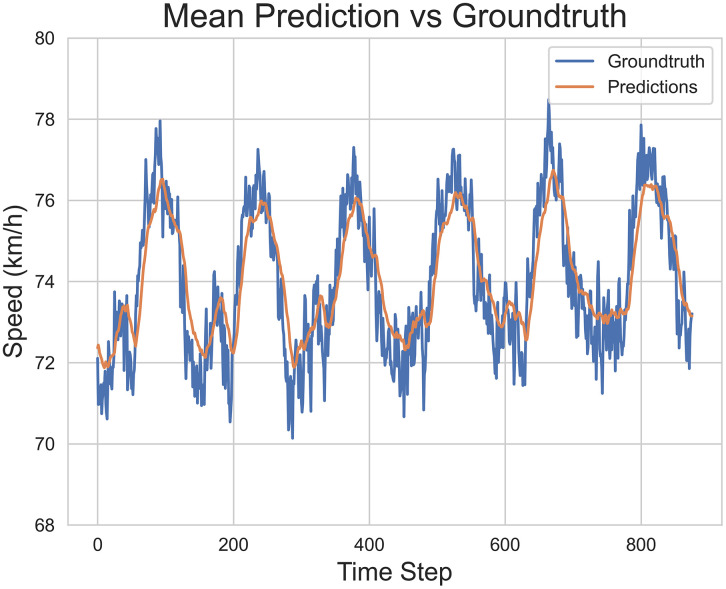
Average prediction results of all segments.

**Fig 5:**
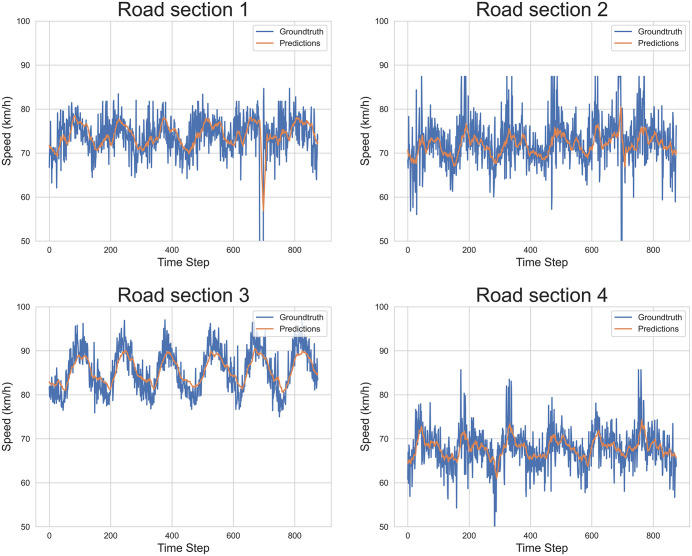
Predicted results of some segments.

To further evaluate predictive performance, Figs 4-5 present the average prediction results of the DBGCN model across all 164 road segments and its hourly prediction performance on the top four selected segments, respectively. The DBGCN prediction curve closely aligns with the actual traffic state trends, maintaining high accuracy even during abrupt traffic state changes. This demonstrates that the model effectively captures the spatiotemporal evolution patterns of macro-level traffic flow.

Another advantage of the proposed DBGCN model is its high interpretability. Specifically, the model can identify source nodes responsible for congestion based on changes in traffic conditions. These source nodes may correspond to construction zones or incident affected sections. Once identified, the model designates them as initial parent nodes and searches for potential child nodes. These child nodes exist within the network in both the current and subsequent time periods, forming parent–child node pairs with their respective parent nodes. Through this iterative process, the model progressively constructs a network level congestion propagation structure, illustrating the flow of congestion information across the network.

**Fig 6:**
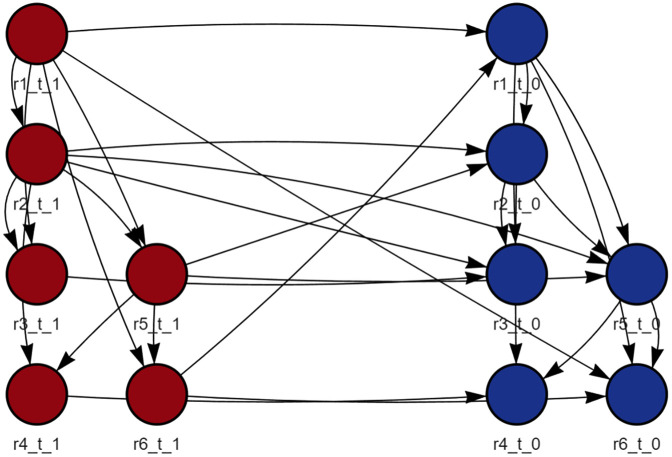
Inference of congestion propagation structure under external influences (partial nodes).

Using a partially combined flow section as an example, Fig 6 illustrates partial inference results from the adjacency-matrix construction during a congestion event. Red nodes represent congestion source nodes at the current time step（t_1）, whereas blue nodes denote potential child nodes at the subsequent time step（t_0）. Arrows indicate propagation paths from parent to child nodes, clearly demonstrating how congestion gradually spreads from the source node（t_1）across the entire network.

## 5 Conclusions

This study addresses the challenge of predicting traffic conditions during road expansion and renovation projects by proposing a Dynamic Bayesian Graph Convolutional Network (DBGCN). The proposed approach integrates dynamic changes in road geometric parameters and traffic organization to achieve accurate real-time traffic conditions forecasting for modified sections.

Experimental results on real highway datasets demonstrate that the proposed DBGCN model significantly outperforms multiple baseline models, including LSTM, DBN, and STGCN, in terms of two key metrics: mean absolute error (MAE) and root mean square error (RMSE). Specifically, the DBGCN model achieved the lowest MAE (2.4112) and RMSE (3.8017), demonstrating a distinct advantage in prediction accuracy.

Furthermore, by incorporating a whitelist–blacklist mechanism to integrate prior knowledge of traffic organization into the graph structure learning process, the proposed DBGCN model adapts to changes in road geometry and traffic organization. This enables the model to generate dynamic adjacency matrices that align with the current construction environment. This capability provides the model with enhanced robustness and flexibility for predicting traffic flow in complex and dynamic scenarios.

Nevertheless, it should be noted that the proposed DBGCN model relies on planned traffic organization schemes to construct the whitelist and blacklist. In practice, the implementation of traffic organization is subject to stochasticity caused by unpredictable factors such as severe weather, equipment failures, or on site logistical delays.

In summary, the DBGCN model proposed in this study provides an effective solution for predicting traffic conditions on road sections undergoing expansion and renovation. By integrating road alignment metrics with dynamic traffic organization information, the DBGCN model not only enhances prediction accuracy but also improves the capacity to represent complex traffic environments. Future research could explore incorporating additional external factors (e.g., weather conditions, incident data) into the model to further enhance prediction performance. In particular, future work may investigate the integration of real-time incident reports and meteorological data to dynamically calibrate the adjacency matrix, thereby enhancing the model’s robustness against unplanned disruptions. In addition, optimizing the model structure and parameter settings to improve computational efficiency warrants further investigation. Overall, this study provides new insights and technical support for intelligent traffic management and urban planning.

## Supporting information

S1 File Road geometric data.csv. Road length, location, number of lanes, etc.
(CSV)

S2 File Speed limit.csv. Speed limit for each road.
(CSV)

S3 File Speed part 1.csv. Speed data for each road segment. The file is uploaded in separate parts.
(CSV)

S4 File Speed part 2.csv. Speed data for each road segment. The file is uploaded in separate parts.
(CSV)

S5 File Speed part 3.csv. Speed data for each road segment. The file is uploaded in separate parts.
(CSV)

S6 File Speed part 4.csv. Speed data for each road segment. The file is uploaded in separate parts.
(CSV)

S7 File Speed part 5.csv. Speed data for each road segment. The file is uploaded in separate parts.
(CSV)
